# *Streptococcus mutans* Can Modulate Biofilm Formation and Attenuate the Virulence of *Candida albicans*

**DOI:** 10.1371/journal.pone.0150457

**Published:** 2016-03-02

**Authors:** Júnia Oliveira Barbosa, Rodnei Dennis Rossoni, Simone Furgeri Godinho Vilela, Janaína Araújo de Alvarenga, Marisol dos Santos Velloso, Márcia Cristina de Azevedo Prata, Antonio Olavo Cardoso Jorge, Juliana Campos Junqueira

**Affiliations:** 1 Department of Biosciences and Oral Diagnosis, Univ Estadual Paulista/UNESP, São José dos Campos, São Paulo, Brazil; 2 Embrapa Gado de Leite, Juiz de Fora, Minas Gerais, Brazil; Institute of Microbiology, SWITZERLAND

## Abstract

*Streptococcus mutans* and *Candida albicans* are found together in the oral biofilms on dental surfaces, but little is known about the ecological interactions between these species. Here, we studied the effects of *S*. *mutans* UA159 on the growth and pathogencity of *C*. *albicans*. Initially, the effects of *S*. *mutans* on the biofilm formation and morphogenesis of *C*. *albicans* were tested *in vitro*. Next, we investigate the influence of *S*. *mutans* on pathogenicity of *C*. *albicans* using *in vivo* host models, in which the experimental candidiasis was induced in *G*. *mellonella* larvae and analyzed by survival curves, *C*. *albicans* count in hemolymph, and quantification of hyphae in the host tissues. In all the tests, we evaluated the direct effects of *S*. *mutans* cells, as well as the indirect effects of the subproducts secreted by this microorganism using a bacterial culture filtrate. The *in vitro* analysis showed that *S*. *mutans* cells favored biofilm formation by *C*. *albicans*. However, a reduction in biofilm viable cells and inhibition of hyphal growth was observed when *C*. *albicans* was in contact with the *S*. *mutans* culture filtrate. In the *in vivo* study, injection of *S*. *mutans* cells or *S*. *mutans* culture filtrate into *G*. *mellonella* larvae infected with *C*. *albicans* increased the survival of these animals. Furthermore, a reduction in hyphal formation was observed in larval tissues when *C*. *albicans* was associated with *S*. *mutans* culture filtrate. These findings suggest that *S*. *mutans* can secrete subproducts capable to inhibit the biofilm formation, morphogenesis and pathogenicity of *C*. *albicans*, attenuating the experimental candidiasis in *G*. *mellonella* model.

## Introduction

The oral cavity is colonized with different microbial species that are usually organized in biofilms adhered to a solid surface such as dental enamel, root surface, or dental implants. An interesting characteristic of biofilms is the presence of a wide variety of microbial species and the interactions between these microorganisms [1–3]. Despite the abundant interactions between fungi and bacteria in the oral cavity, our knowledge of the mechanisms involved in these interactions is still limited. The elucidation of the interaction mechanisms between different microbial species is extremely important for the understanding of the pathogenesis of human diseases. Furthermore, knowledge of the natural mechanisms whereby microorganisms compete with each other and establish antagonistic interactions may contribute to the discovery of new therapeutic strategies for human infections [4].

*Candida albicans* is a human pathogen that, in addition to oral candidiasis, can cause various polymicrobial diseases due to its ability to form multispecies biofilms. In this respect, the ecological interactions between yeast of the genus *Candida* and different bacterial species found in the oral cavity, such as *Streptococcus mutans*, have become the subject of interest in scientific studies. Pereira-Cenci et al. [5] observed that *S*. *mutans* (UA159) stimulated the growth of *C*. *albicans* in *in vitro* biofilms, but suppressed the formation of hyphae by this yeast. On the basis of these results, Jarosz et al. [1] evaluated the interaction between *S*. *mutans* UA159 and *C*. *albicans* based on production of quorum sensing molecules. Filter sterilized spent medium of *S*. *mutans* inhibited germ tube formation by *C*. *albicans* indicating that *S*. *mutans* secretes one or more diffusible molecules that affect hypha formation by *C*. *albicans*. Next, Joyner et al. [6] attributed the inhibitory effects on *C*. *albicans* morphogenesis to a natural peptide produced by *S*. *mutans*, which was called mutanobactin A.

All of these studies on the interaction between *S*. *mutans* and *C*. *albicans* cited earlier have used *in vitro* biofilm models. *In vivo* studies are becoming increasingly limited due to ethical issues to the use of rats or mice in scientific research. More recently, invertebrate models of infection, such as *Galleria mellonella*, are being developed because of their numerous advantages compared to mammalian models, including their low cost, easy handling and the possibility of large-scale studies. Additionally, these models have no ethical restrictions and can be used as a screening tool for studies using vertebrate models, thus reducing the number of rats or mice necessary [7–11]. *G*. *mellonella* has been successfully used in the medical field as a model for the study of *Candida* pathogenesis, since it permits the injection of a standardized inoculum of *C*. *albicans* and contains different types of hemocytes and antimicrobial peptides that play an important role in the defense against pathogens [7, 12–16].

Since the previous studies showed that *S*. *mutans* produces signaling molecule capable to inhibit *C*. *albicans* cultured *in vitro* and no study was conducted to investigate the activity of *S*. *mutans* on the candidiasis development, the objective of the present study was to evaluate the effects of *S*. *mutans* on biofilm formation and morphogenesis of *C*. *albicans in vitro* and expand these findings for *in vivo* studies using *G*. *mellonella* as a model of experimental candidiasis.

## Materials and Methods

### Inoculum preparation of *C*. *albicans* and *S*. *mutans*

In this study, we used reference strains of *Candida albicans* (ATCC 18804) and *Streptococcus mutans* (UA159) maintained in a freezer at -80°C in the Laboratory of Microbiology and Immunology, Institute of Science and Technology, UNESP, Campus of São José dos Campos, Brazil. *C*. *albicans* was cultured for 18 h at 37°C in yeast nitrogen base broth (YNB; Difco, Detroit, USA) supplemented with 100 mM glucose. *S*. *mutans* was grown in brain-heart infusion broth (BHI; Difco, Detroit, USA) supplemented with 5% sucrose for 4 h at 37°C in a bacteriological incubator under a partial pressure of 5% CO_2_. Cells were collected by centrifugation and washed three times with phosphate-buffered saline (PBS). Suspensions of each microorganism were standardized in PBS at a concentration of 10^7^ cells/mL using a spectrophotometer (B582, Micronal, São Paulo, Brazil). Cells densities of the inoculum were confirmed by CFU/mL counting after plating in Sabouraud dextrose agar for *C*. *albicans* and BHI agar for *S*. *mutans*.

### Preparation of the *S*. *mutans* culture filtrate

Firstly, the standardized suspension of *S*. *mutans* containing 10^7^ cells/mL was prepared as described above. A volume of 1 mL of the standardized suspension was transferred to a Falcon tube containing 6 mL of brain-heart infusion broth supplemented with 5% sucrose and the mixture was incubated for 4 h at 37ºC under a partial pressure of 5% CO_2_. After this period, the culture was centrifuged and the supernatant was filtered through a membrane with a pore diameter of 0.22 μm (MFS, Dublin, USA).

### *In vitro* biofilm formation

The method of biofilm formation on the bottom of a 96-well microtiter plate (Costar Corning, New York, USA) was used according to described by Thein et al. [17]. Firstly, 100 μL of the standardized suspension of *C*. *albicans* was added to each well of the plate. The plate was incubated for 90 min at 37°C under shaking at 75 rpm (Quimis, Diadema, São Paulo, Brazil) to promote initial adhesion of the microorganisms. The suspension was then aspirated and each well was washed two times with PBS to remove weakly adhered cells. Next, 100 μL fetal bovine serum was added to each well and the plates were incubated again for 2 h under shaking. The wells were washed two times with PBS and 50 μL of the standardized *S*. *mutans* suspension or 50 μL of the *S*. *mutans* culture filtrate was added to each well. In the control groups, 50 μL of PBS (Control PBS) or 50 μL of BHI + 5% sucrose were performed (Control BHI). In addition, a control group formed only by 50 μL of the standardized *S*. *mutans* suspension (without *C*. *albicans*) was added.

For biofilm growth and maintenance, 140 μL yeast nitrogen base (YNB) supplemented with 100 mM glucose and 60 μL BHI broth supplemented with 5% sucrose were added in each well. The media were changed at intervals of 24 h and the plates were incubated at 37°C under shaking at 75 rpm for 48 h.

### Analysis of biofilm formation by CFU/mL count

After 48 h of incubation, the wells were washed two times with PBS. Next, 250 μL PBS was added to each well and the biofilm adhered to the bottom of the plate was disrupted by homogenization for 30 s in an ultrasonic homogenizer (Sonics Vibra Cell) at an amplitude of 25%. Serial dilutions were prepared from the solution obtained and 100-μL aliquots were seeded onto Sabouraud dextrose agar and incubated at 37°C. In the groups containing *S*. *mutans* cells, the serial dilutions were seeded onto Mitis Salivarius Bacitracin Sucrose (MSBS) agar and incubated at 37°C under a partial pressure of 5% CO_2_. After 48 h, the number of colony-forming units (CFU) was determined. The study was supported by two experiments at different times with ten biofilms per group.

### Analysis of biofilm formation by total biomass quantification

After biofilm formation as described above, the biofilm biomass was quantified based on the crystal violet (CV) assay described by Peeters et al. [18] with modifications. For fixation of the biofilms, 100 μl of 99% methanol was added (Sigma-Aldrich, São Paulo, Brasil). After 15 min, supernatants were removed and the plates were air-dried. Then, 100 μl of a 1% CV solution was added to all wells. After 20 min, the excess CV was removed by washing with PBS. Finally, bound CV was released by adding 150 μl of 33% acetic acid (Sigma-Aldrich, São Paulo, Brasil). The absorbance was measured at 540 nm. All steps were carried out at room temperature. The CV assay was performed in two independent experiments with six biofilms per group.

### Analysis of biofilm formation by scanning electron microscopy (SEM)

Acrylic resin discs measuring 8 mm in diameter were placed on a 24-well plate for biofilm formation as previously cited. After biofilm formation, the specimens were fixed in 1 mL 2.5% glutaraldehyde for 1 h. The specimens were then dehydrated in an increasing ethanol series (10, 25, 50, 75 and 90%) for 20 min each, followed by immersion in 100% alcohol for 1 h. The plates were kept in an oven at 37°C for 24 h to permit complete drying of the specimens.

After drying, the specimens were transferred to aluminum stubs and sputtered with gold for 160 s at 40 mA (Denton Vacuum Desk II). The specimens were examined and photographed under a JEOL JSM5600 scanning electron microscope at the National Institute for Space Research (Instituto Nacional de Pesquisas Espaciais—INPE). These experiments were performed at two different times with three biofilms per group.

### Induction of *in vitro* filamentation by *C*. *albicans*

For the study of *in vitro* filamentation, the following groups were evaluated: PBS control (*C*. *albicans* + PBS), BHI control (*C*. *albicans* + BHI broth), cell interaction (*C*. *albicans* + *S*. *mutans* cells), and supernatant interaction (*C*. *albicans* + *S*. *mutans* supernatant). In a 24-well culture plate (Costar Corning, New York, USA), 1 mL of distilled water was mixed with 10% fetal bovine serum and 100 μL of the standardized *C*. *albicans* suspension.

According to the experimental group, 50 μL standardized *S*. *mutans* suspension or 50 μL *S*. *mutans* culture supernatant were also added. In the control groups, 50 μL PBS or BHI broth were added to each well. The plates were incubated at 37°C under a partial pressure of 5% CO_2_. Five assays were used per group and the experiment was performed independently in duplicate.

After 24 h of incubation, 50 μL of the inoculum were transferred to glass slides with 10 previously demarcated fields on the back of the slide and observed under a light microscope at 400x magnification. The images were analyzed regarding *C*. *albicans* morphology and 10 microscopic fields per slide were chosen for the quantification of hyphae. According to Vilela et al. [19] the following scores were attributed for the number of hyphae present in each microscopic field: 0 (no hyphae), 1 (1–3 hyphae), 2 (4–10 hyphae), 3 (11–20 hyphae), and 4 (more than 20 hyphae).

### Experimental candidiasis in the *Galleria mellonella* model

The methodology described by Mylonakis et al. [20] and Fuchs et al. [7] was used in this study. *Galleria mellonella* (Embrapa Gado de Leite, Juiz de Fora, MG, Brazil) in the final stage of the larval phase and weighing approximately 250 mg were stored in the dark and used within 7 days from shipment. The larvae were kept without food throughout the experiment.

Before the study of the interaction between *C*. *albicans* and *S*. *mutans*, the susceptibility of *G*. *mellonella* to infection with *S*. *mutans* was analyzed to determine the sublethal concentration of this microorganism in these animals. Standardized suspensions containing different concentrations (10^4^ to 10^7^ cells/larva) of *S*. *mutans* were injected into *G*. *mellonella* and the survival curve was determined. The suspensions were standardized in a spectrophotometer as described above and a group of 16 larvae was used per concentration. These experiments were performed independently in duplicate.

For the study of *C*. *albicans* and *S*. *mutans* interaction, standardized suspensions of *C*. *albicans* (10^6^ cells/larva) were injected into the hemolymph of each larva through the last left proleg using a Hamilton syringe (Hamilton, Inc., Reno, USA). Next, standardized suspensions of *S*. *mutans* containing 10^5^ cells/larva (sublethal concentration for *G*. *mellonella* as defined in the previous test) or *S*. *mutans* supernatant were inoculated into the right proleg. A group inoculated only with PBS was used to show that death was not due to needle trauma. Another non-injected group was included to monitor the health status of the *G*. *mellonella* larvae throughout the experiment.

### *G*. *mellonella* survival assays

After microbial injections, the larvae were incubated at 37°C in a bacteriological oven and observed 18, 24, 48, 72, 96, 120, 144 and 168 hours after infection. The number of dead *G*. *mellonella* was recorded daily. The larvae were considered to be dead when they showed no sign of movement after touch. Sixteen larvae were used per experimental group. These experiments were performed independently in duplicate.

### CFU/mL count of *C*. *albicans* in the hemolymph of *G*. *mellonella*

To quantify the presence of *C*. *albicans* in *G*. *mellonella* infection, samples of larval hemolymph were removed at 0, 8 and 12 h after injections. Hemolymph was collected from a pool of three larvae per time point and experimental group, which is sufficient to prepare serial dilutions. The experiment was carried out in duplicate using 9 larvae per group.

The larvae were cut with a scalpel blade in the ventral part and gently squeezed to remove the hemolymph. Serial dilutions were prepared from 10 μL of collected hemolymph and seeded onto Sabouraud dextrose agar containing 100 mg/L chloramphenicol for the growth of *C*. *albicans*. The plates were incubated for 48 h at 37°C and colonies were counted for the calculation of CFU/mL.

### *G*. *mellonella* histological analysis

Histological analysis was performed to determine the effects of *S*. *mutans* on *C*. *albicans* filamentation in *G*. *mellonella*. Eighteen hours after inoculation, the fat body and other internal structures of *G*. *mellonella* was removed with a scalpel blade, stored in 10% formalin, and sent for histological processing. Histological sections (5 μm) were mounted on glass slides and stained with periodic acid Schiff (PAS). The yeast and hyphal forms of *C*. *albicans* present in the internal tissues of *G*. *mellonella* were observed under a light microscope. For analysis of filamentation, all areas stained with PAS, indicating the presence of yeast cells and hyphae, were photographed with a Cyber-Shot DSC-585 digital camera (Sony Corporation) coupled to a Zeiss Axiophot 2 light microscope (Carl Zeiss, Oberkochen, Germany) at 100x magnification. The area (μm) occupied by yeast cells and hyphae was determined in each image using the Image J program (version 1.32 for Windows, National Institutes of Health/NIH, Bethesda, USA) according to Vilela et al. [19]. In each histological section, the delimited areas were summed and Log_10_ transformed. Five larvae were used per group.

### Statistical analysis

Analysis of variance (ANOVA) and the Tukey test were used for the CFU/mL and biomass quantification of the *in vitro* biofilm formation tests, to analyze the presence of *C*. *albicans* in *G*. *mellonella* hemolymph, and for histological analysis of *G*. *mellonella*. The scores obtained by the analysis of *in vitro* filamentation were compared by the Kruskal-Wallis and Dunn’s test. A survival curve was constructed to analyze the survival of *G*. *mellonella* and differences were estimated by the log-rank method (Mantel-Cox). All analyses were performed using the Graph Pad Prism 5 Program and a level of significance of 5% was adopted.

## Results

### Effects of *S*. *mutans* on biofilm formation by *C*. *albicans* (*in vitro* study)

**Fig 1:**
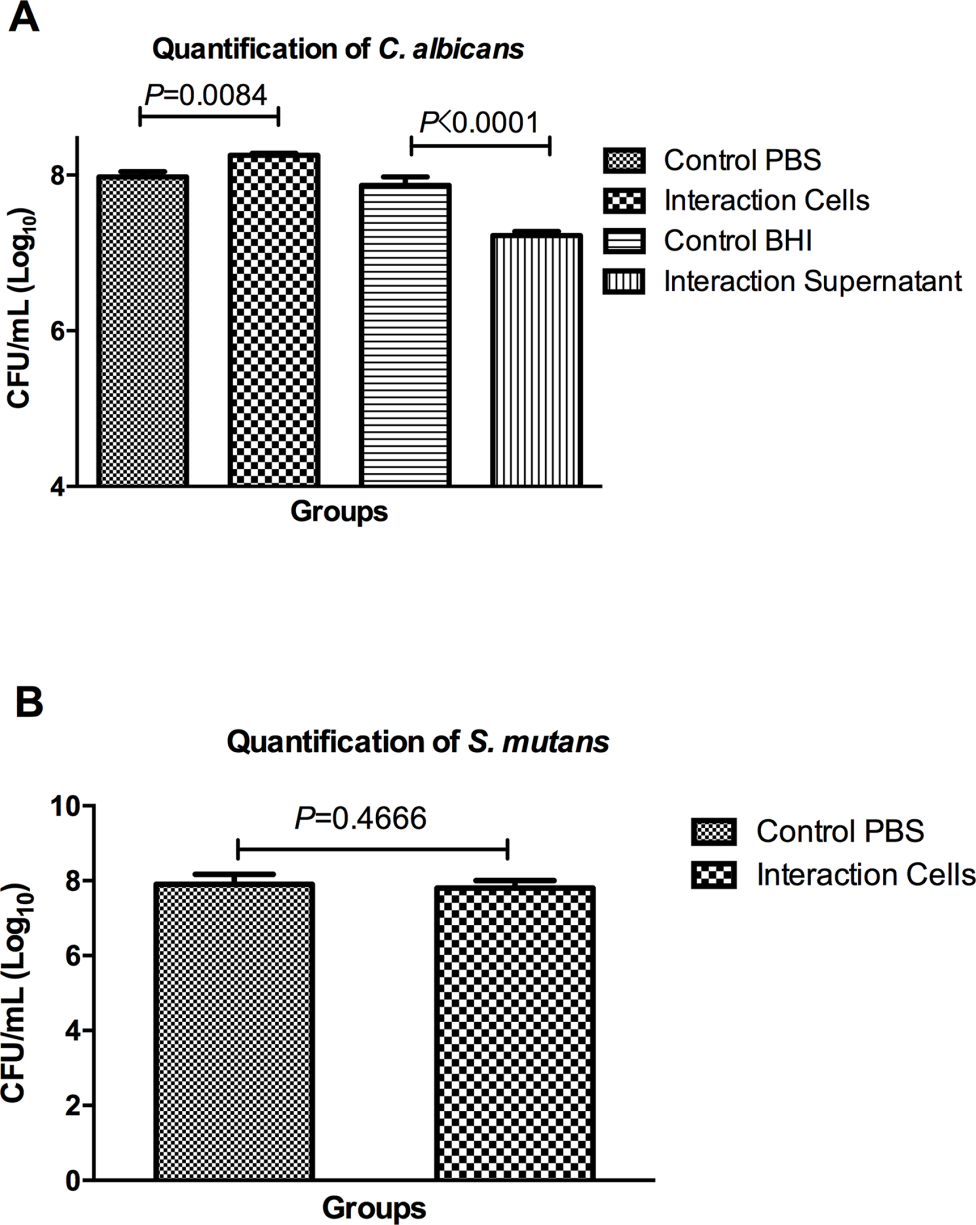
Quantitative analysis of the *in vitro* biofilm formation by the CFU/mL count: A) Mean and standard deviation of the number of *C*. *albicans* CFU/mL (Log_10_) for the following groups: biofilms formed by *C*. *albicans* and PBS (Control group), mixed biofilms formed by *C*. *albicans* and *S*. *mutans* cells (Cells interaction group), biofilms formed by *C*. *albicans* and BHI (Control group) and biofilms formed by *C*. *albicans* and *S*. *mutans* supernatant filtrate (Supernatant interaction group). B) Mean and standard deviation of the number of *S*. *mutans* CFU/mL (Log_10_) for the groups: single biofilms formed by *S*. *mutans* and PBS (Control group), mixed biofilms formed by *C*. *albicans* and *S*. *mutans* cells (Cells interaction group). Tukey test, *P* ≤ 0.05.

Analysis of the interaction between *S*. *mutans* and *C*. *albicans* in the *in vitro* model of biofilm formation showed a higher *C*. *albicans* count (CFU/mL) in the mixed biofilms formed by *C*. *albicans* and *S*. *mutans* cells compared to the single biofilms formed by *C*. *albicans* and PBS (Control group). However, a reduction in *C*. *albicans* counts was observed when the biofilms of *C*. *albicans* were associated with the *S*. *mutans* culture supernatant (**Fig 1A**). These data indicate that the number of *C*. *albicans* in the biofilms were stimulated by the *S*. *mutans* cells and inhibited by the *S*. *mutans* supernatant.

In the group with mixed biofilms formed by *C*. *albicans* and *S*. *mutans* cells, the number of *S*. *mutans* was also determined (**Fig 1B**). The mixed biofilms showed similar results of CFU/mL compared to the single biofilm formed only by *S*. *mutans* (without *C*. *albicans*).

**Fig 2:**
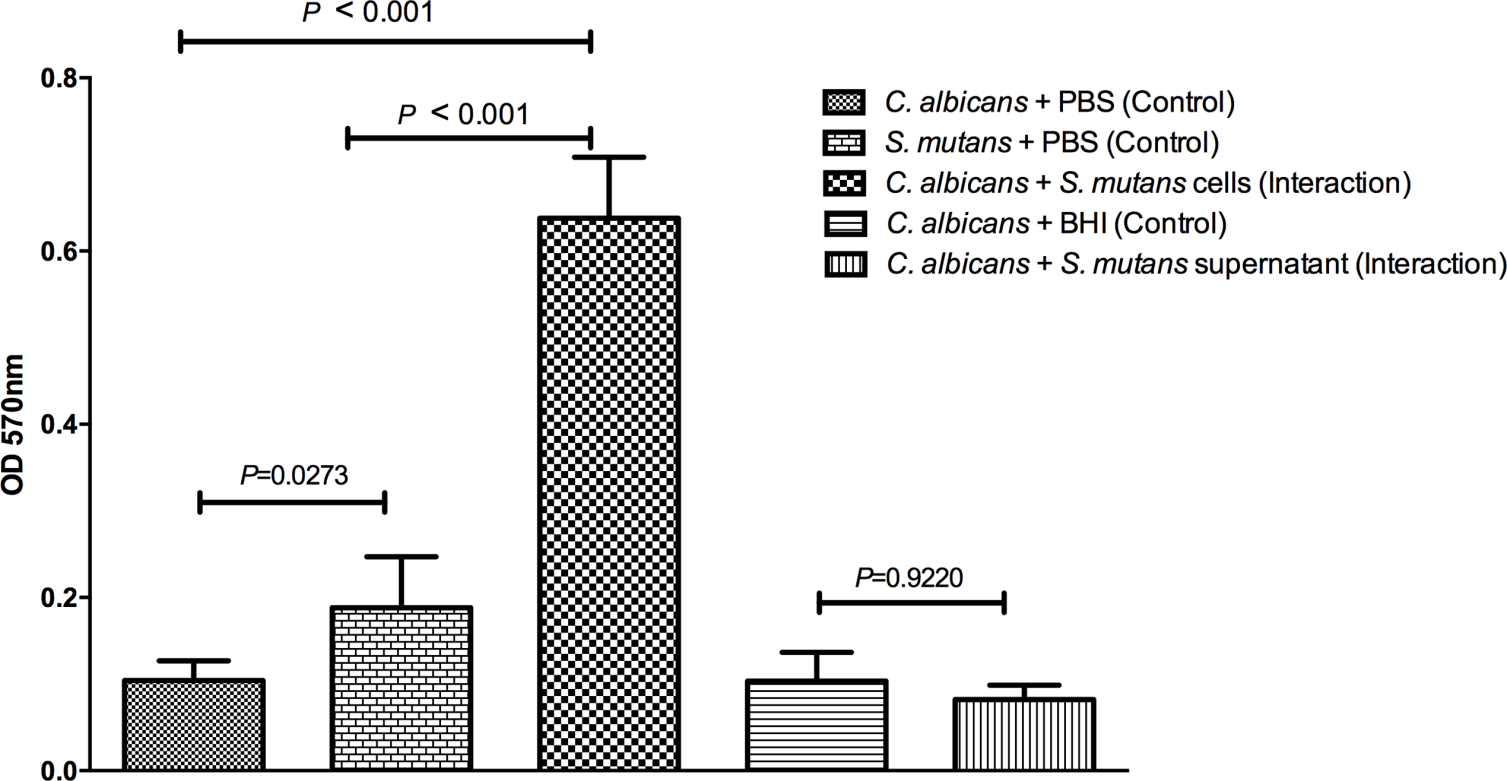
Mean and standard deviation of the biofilm mass obtained in the crystal violet assay for the following groups: biofilms formed by *C*. *albicans* + PBS (Control group); biofilms formed by *S*. *mutans* + PBS (Control group); mixed biofilms formed by *C*. *albicans* + *S*. *mutans* cells (Interaction group); biofilms formed by *C*. *albicans* + BHI (Control group); and biofilms formed by *C*. *albicans* and *S*. *mutans* supernatant filtrate (Interaction group). Tukey test, *P* ≤ 0.05.

In the crystal violet assay, the biofilms formed by *C*. *albicans* and *S*. *mutans* cells (Interaction group) exhibited a significant increase of the total biomass compared to the control groups formed only by *C*. *albicans* or *S*. *mutans*. In relation to the indirect effects of *S*. *mutans* on *C*. *albicans*, the presence of *S*. *mutans* supernatant was not able to reduce the total biofilm formed by *C*. *albicans* (**Fig 2**). These results show that *S*. *mutans* supernatant can cause reduction of *C*. *albicans* viable cells (CFU/mL), but it is not enough to reduce the total biomass of *C*. *albicans* biofilm.

**Fig 3:**
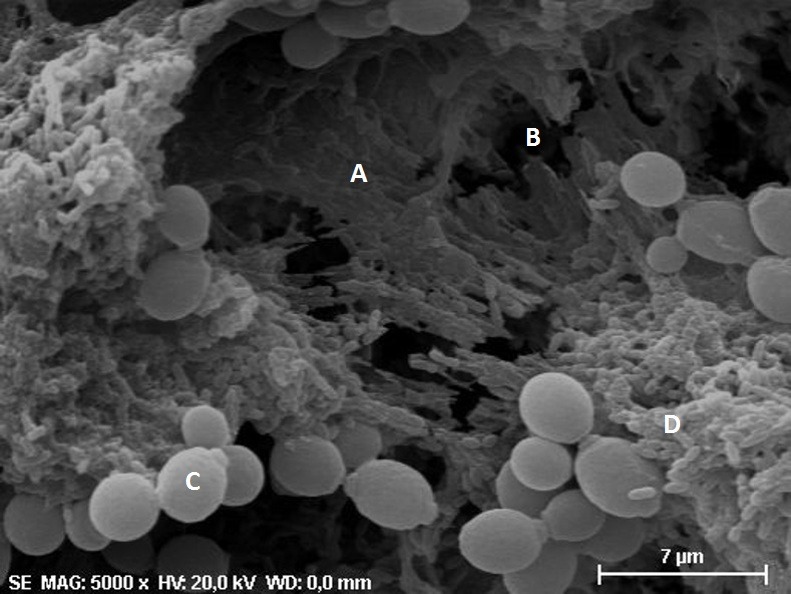
Scanning electron microscopy of the mixed biofilm formed by *C*. *albicans* and *S*. *mutans* cells, indicating the presence of a mature biofilm formed by polymeric extracellular matrix (A), water channel (B), *C*. *albicans* yeast cell (C), and *S*. *mutans* cell (D). Original magnification: 5000x.

**Fig 4:**
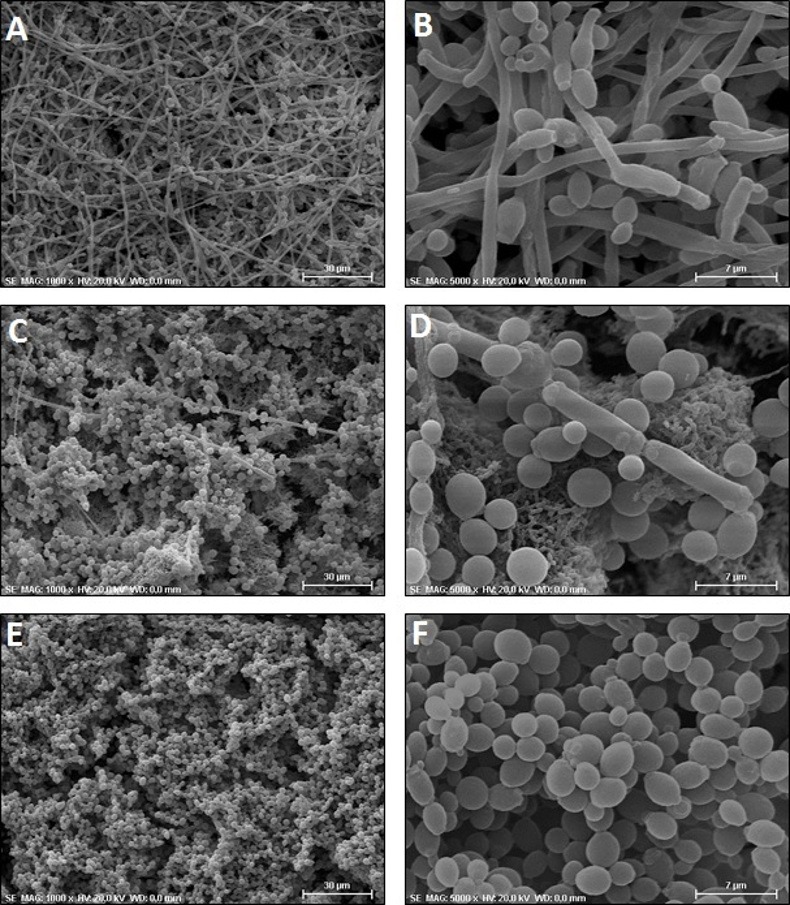
Scanning electron microscopy of the biofilms formed *in vitro*. A-B) Biofilms formed by *C*. *albicans* and PBS (Control group): predominance of hyphae and few *C*. *albicans* yeast cells. Original magnification: (A) 1000x and (B) 5000x. C-D) Biofilms formed by *C*. *abicans* and *S*. *mutans* cells (Cells Interaction group): reduction in hyphae formation and the predominance of *C*. *albicans* yeast cells. Original magnification: (C) 1000x and (D) 5000x). E-F) Biofilms formed by *C*. *albicans* and *S*. *mutans* supernatant (Supernatant interaction group): yeast cells with total inhibition of hyphae formation. Original magnification: (E) 1000x and (F) 5000x.

The biofilms formed *in vitro* were also analyzed by scanning electron microscopy (SEM), in which we observed a mature biofilm formation on acrylic resin discs after 48 hours of incubation. The biofilms showed *C*. *albicans* cells, an extracellular polymeric matrix and water channels responsible for biofilm nutrition. In the mixed biofilms, the presence of *S*. *mutans* cells was also confirmed (**Fig 3**). The *C*. *albicans* cells observed in the biofilms showed morphological variations according to the experimental group. In the biofilms formed by *C*. *albicans* and PBS (Control group), we verified a large number of hyphae in contrast with a few yeast cells (**Fig 4A and 4B**). However, we found a predominance of yeast cells with a reduction in the hyphae formation in the biofilms formed by *C*. *albicans* and *S*. *mutans* cells (**Fig 4C and 4D**) and a total absence of hyphae in the biofilms formed by *C*. *albicans* and *S*. *mutans* supernatant (**Fig 4E and 4F**), indicating that the *S*. *mutans* inhibited the hyphae formation by *C*. *albicans* in the biofilms.

### Effects of *S*. *mutans* on *C*. *albicans* filamentation (*in vitro* study)

**Fig 5:**
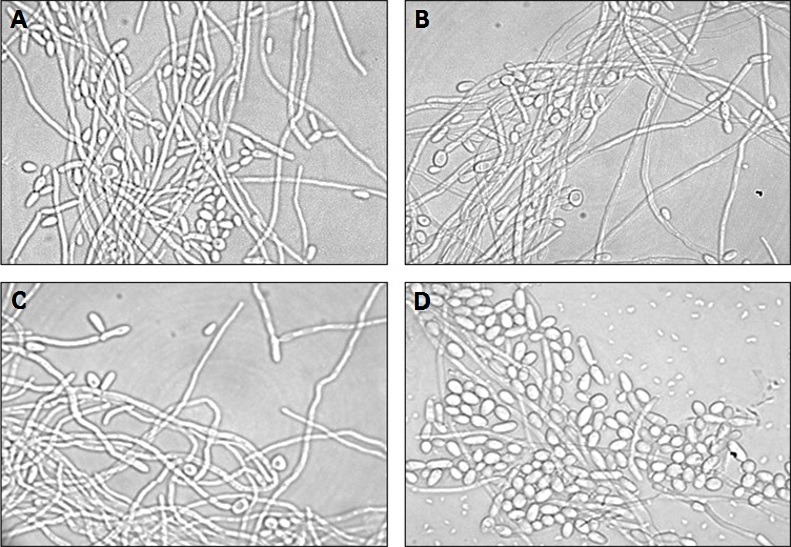
Light microscopy photomicrographs illustrating the morphology of *C*. *albicans* when in contact with: A) PBS (control group); B) *S*. *mutans* cells (cell interaction group); C) BHI broth (control group); D) *S*. *mutans* culture supernatant (supernatant interaction group). In the picture D, we can observe a predominance of yeast cells compared to the other groups in which hyphae predominated (A, B and C), demonstrating that the *S*. *mutans* supernatant interfered with the morphological transition of *C*. *albicans* inhibiting hyphal formation

**Fig 6:**
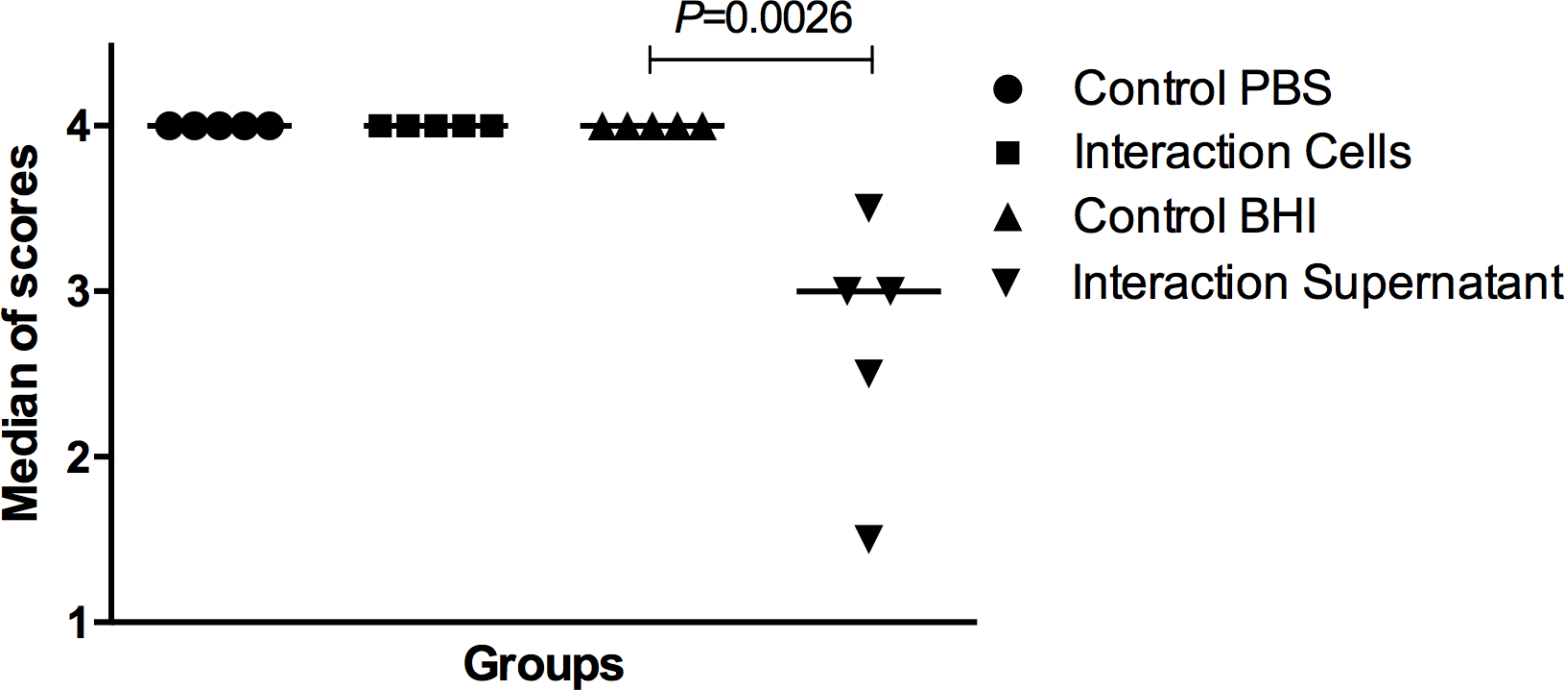
Median scores obtained by determining the number of hyphae in the in vitro C. albicans filamentation assay for the following groups: Control group with PBS; Interaction group with S. mutans cells; Control group with BHI broth; Interaction group with S. mutans supernatant. The scores were attributed according to the number of hyphae in each microscopic field: 0 (no hyphae), 1 (1–3 hyphae), 2 (4–10 hyphae), 3 (11–20 hyphae), and 4 (more than 20 hyphae). A significant hyphae reduction was observed in the supernatant interaction group when compared to the Control group with BHI broth. Dunn’s test, *P* ≤ 0.05.

After we verified that *S*. *mutans* inhibited the hyphae formation by *C*. *albicans* in biofilms formed *in vitro*, another experiment focusing in the *C*. *albicans* filamentation was conducted. In this experiment, *C*. *albicans* was placed in a 24-well culture plate containing fetal bovine serum to induce the hyphae formation. The plates were incubated for 24 h and transferred to glass slides for morphological analysis. We observed a large number of *C*. *albicans* hyphae in the following groups: Control with PBS; Control with BHI; and Interaction with *S*. *mutans* cells. The presence of *S*. *mutans* cells was not capable to alter hyphae formation by *C*. *albicans*. However, we verified a significant inhibition of the hyphae formation when *C*. *albicans* was incubated in the presence of the *S*. *mutans* supernatant compared to the control groups (PBS or BHI broth) (**Figs 5 and 6**).

Besides bovine serum, another factor that plays an important role in filamentation is the pH of the medium. Therefore, the pH of the culture medium was measured. The pH of the *S*. *mutans* supernatant was 7.0 and the BHI broth supplemented with 5% sucrose was 7.3. These data show that the inhibition of *C*. *albicans* filamentation by *S*. *mutans* supernatant cannot be attributed to pH variations in the culture medium.

Taken together, the *in vitro* results obtained in this study suggest that *S*. *mutans* secretes subproducts into the culture medium, which exert inhibitory effects on *C*. *albicans*, interfering with the number of viable cells in the biofilm formation and filamentation capacity. In order to determine whether *S*. *mutans* also exerts inhibitory effects on the pathogenesis of *C*. *albicans*, we extended this study to an *in vivo* host model.

### Effects of *S*. *mutans* on experimental candidiasis: *G*. *mellonella* survival curve

First, we evaluated the susceptibility of *G*. *mellonella* to infection with different concentrations of *S*. *mutans* (10^4^ to 10^7^ cells/larva) to determine the sublethal concentration of this microorganism in these animals. The results showed that *S*. *mutans* pathogenicity was dose-dependent in *G*. *mellonella*; concentrations of 10^6^ and 10^7^ *S*. *mutans* cells/larva resulted in mortality rates of 20 and 30% in the end of the experiment, whereas concentrations of 10^4^ and 10^5^ cells/larva did not cause lethal infection in these animals (S1 Fig). Thus, the sublethal concentration of 10^5^ cells/larva was adopted for the subsequent assays.

**Fig 7:**
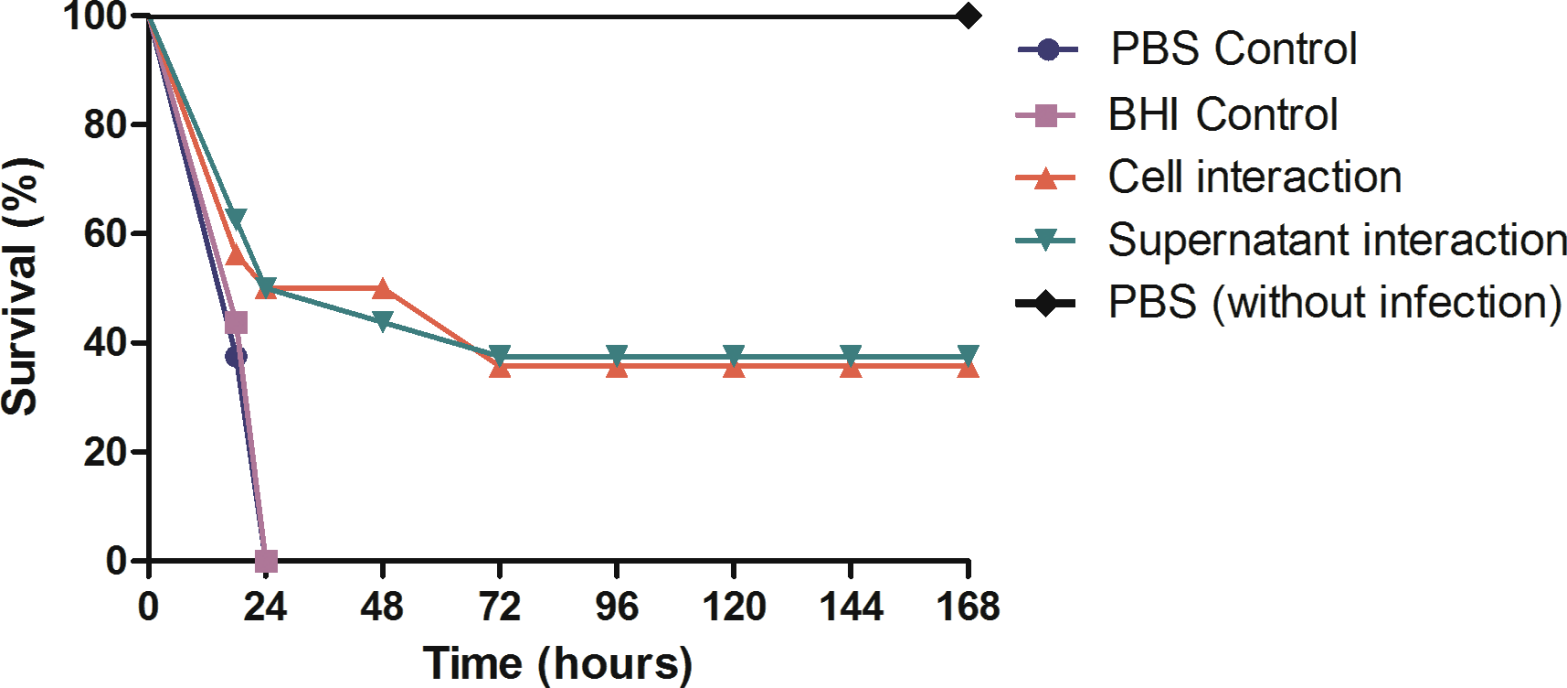
Survival curves of *G*. *mellonella* larvae in the groups inoculated with: *C*. *albicans* and PBS (control group); *C*. *albicans* and BHI broth (control group); *C*. *albicans* and *S*. *mutans* cells (cells interaction group); *C*. *albicans* and *S*. *mutans* culture supernatant (supernatant interaction group); only PBS (without infection). Statistically significant differences were observed between the cells interaction group and control group with PBS (*P* = 0.0068) and between the supernatant interaction group and control group with BHI broth (*P* = 0.0043). Log-rank test, *P* ≤ 0.05.

After that, we moved on to the study of the effects of *S*. *mutans* on *C*. *albicans* pathogenicity in the *G*. *mellonella* larvae. Analysis of the survival curve of *G*. *mellonella* showed that the pathogenicity of *C*. *albicans* in *G*. *mellonella* varied according to the group studied. The groups infected by *C*. *albicans* and inoculated only with PBS or BHI broth (Control groups) resulted in death of 100% of the larvae within 24 h after infection. However, the larval survival rate increased significantly when the infection by *C*. *albicans* in *G*. *mellonella* was followed by the injection of *S*. *mutans* cells or supernatant (Interaction groups) **(Fig 7)**. These findings indicate that *S*. *mutans* was able to attenuate the infection caused by *C*. *albicans* in *G*. *mellonella* model. Then, we investigated the effects of *S*. *mutans* on the number of *C*. *albicans* present in the hemolymph and in the internal tissues of *G*. *mellonella* using, respectively, assays of CFU/mL count and histological analysis.

### Effects of *S*. *mutans* on experimental candidiasis: CFU/mL count of *C*. *albicans* in the hemolymph of *G*. *mellonella*

The study of *G*. *mellonella* hemolymph culture revealed a similar growth pattern of *C*. *albicans* in all groups at the different time points studied. Immediately after infection with *C*. *albicans* (time 0), the number of CFU/mL in the hemolymph was approximately 6 Log, decreasing to 4–5 Log after 8 h and again increasing to 6 Log after 12 h (S2 Fig). In this experiment, statistically significant differences were not observed between the interaction and control groups.

### Effects of *S*. *mutans* on experimental candidiasis: *G*. *mellonella* histological analysis

**Fig 8:**
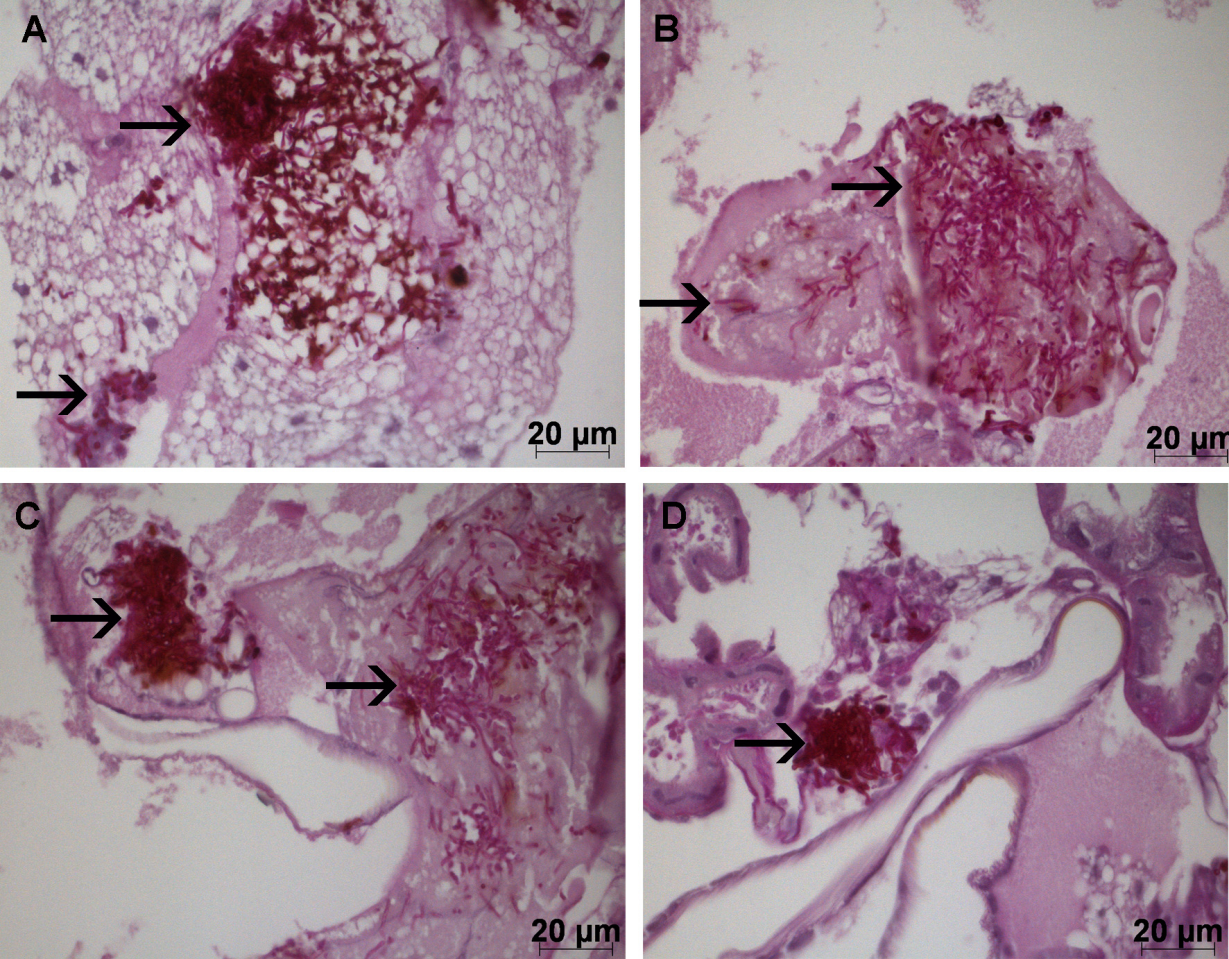
Histological images of the yeast and hyphae (➔) in the fat body and other internal structures of *G*. *mellonella*. A) Control group with PBS. B) Interaction group with *S*. *mutans* cells. C) Control group with BHI broth. D) Interaction group with *S*. *mutans* supernatant.

**Fig 9:**
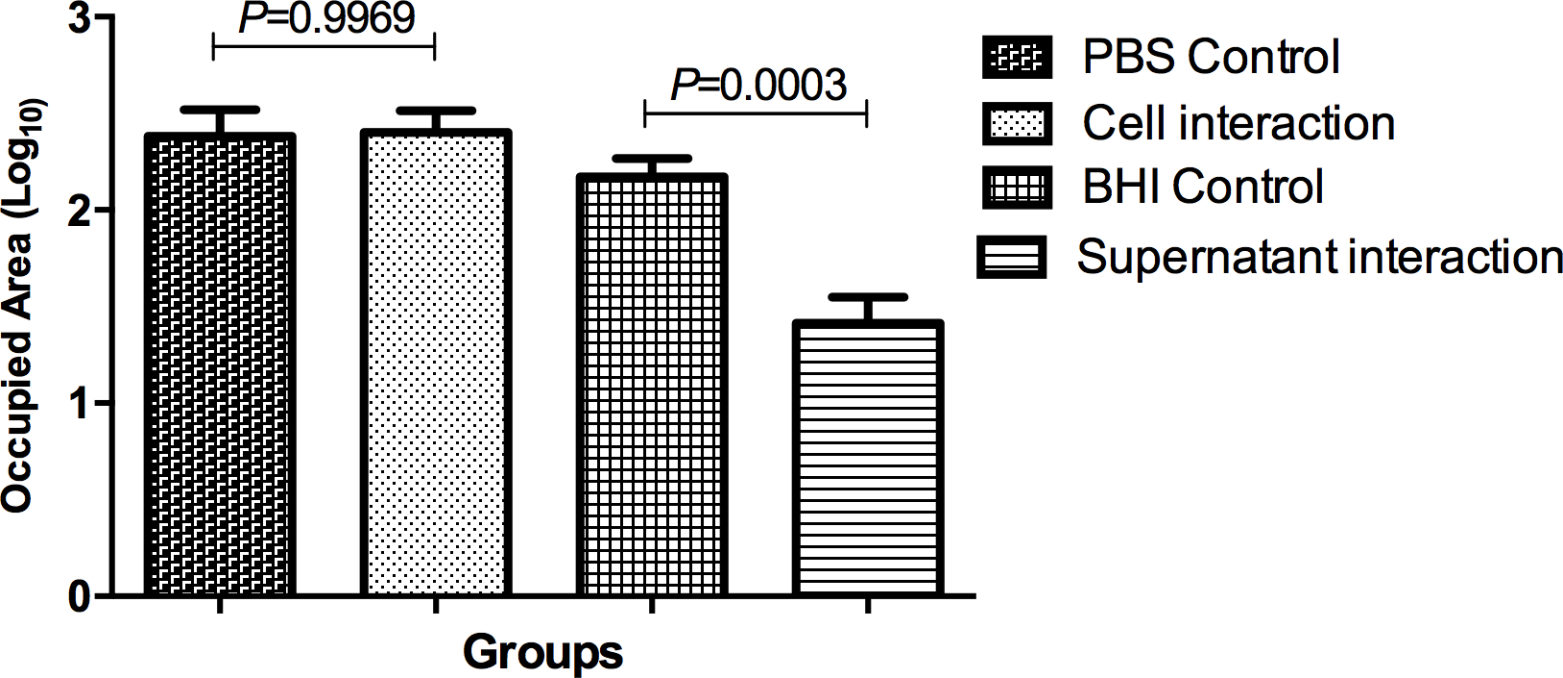
Mean and standard deviation of the area occupied (Log_10_) by hyphae and/or yeast cells after 18 h of infection for the following groups: Control group with PBS; Interaction group with *S*. *mutans* cells; Control group with BHI broth; Interaction group with *S*. *mutans* supernatant. Tukey test, *P* ≤ 0.05.

Histological analysis of the fat body and other internal structures of *G*. *mellonella* showed the presence of extensive aggregations of yeast and hyphae in the tissues of animals infected with *C*. *albicans* **(Fig 8)**. The number of *C*. *albicans* was quantified in all groups. Significant reduction in *Candida* count was only observed in the supernatant interaction group when compared to the control group (**Fig 9**). Therefore, as observed in the *in vitro* tests, the *S*. *mutans* culture supernatant was able to inhibit the morphological transition of *C*. *albicans*.

## Discussion

It has been estimated that 65% of human infections are associated with the formation of biofilms on the surfaces of host tissues or medical devices [21]. Studies have shown that bacteria and fungi present in biofilms can influence each other through the secretion of extracellular signaling molecules or physical interactions of cell-cell contact or aggregation [4]. Since *C*. *albicans* and *S*. *mutans* are found together in the oral biofilms on dental surfaces [22, 23], in this study we investigate the influence of *S*. *mutans* on the growth and pathogencity of *C*. *albicans*.

In an attempt to understand the effect of *S*. *mutans* on the growth of *C*. *albicans* in mixed biofilms, we used three different assays to evaluate the biofilms formed *in vitro*: CFU/mL count, crystal violet assay, and scanning electron microscopy (SEM).

In the CFU/mL analyses, the results showed a higher *C*. *albicans* count in the mixed biofilms formed by *C*. *albicans* and *S*. *mutans* cells compared to the single biofilms formed only by *C*. *albicans*, indicating that *S*. *mutans* cells were capable to stimulate the growth of *C*. *albicans*. It has been described that *S*. *mutans* can improve the growth of *C*. *albicans* cells in mixed biofilms [5, 23]. According to Sztajer et al. [23], *C*. *albicans* can be more efficient than *S*. *mutans* in taking up sucrose. The sucrose has been considered an important factor in the interaction of *C*. *albicans* and *S*. *mutans* in biofilms formed *in vitro*. When sucrose is present in the culture medium, as in our study, the adhesive interaction between these two microorganisms is enhanced [24, 25]. Thein et al. [17] studied mixed biofilms of *S*. *mutans* and *C*. *albicans* in the presence of glucose and no significant effect of *S*. *mutans* on the viability of *C*. *albicans* was found.

Novel assays for quantification of bacteria and fungi in biofilms have been used rather than assays based on the quantification of viable cells (CFU/mL count). Crystal violet assay allows to quantifying the biofilm biomass in the entire well of microtiter plates. This dye binds to negatively charged molecules and polysaccharides, staining both living and dead cells, as well as extracellular matrix [18]. In this study, the mixed biofilms formed by *C*. *albicans* and *S*. *mutans* cells had a significant increase of the total biomass compared to the control groups formed only by *C*. *albicans* or *S*. *mutans*. Although in this study, we focused on the influence of the *S*. *mutans* on *C*. *albicans*, this assay demonstrated that *C*. *albicans* also provides benefits to *S*. *mutans* in mixed biofilms. Falsetta et al. [25] reported that the association between *C*. *albicans* and *S*. *mutans* can be mediated by a physical interaction that relies on the production of glucans, which are produced by bacterial exoenzymes (glucosyltransferases) on yeast and hyphal cell surface. These interactions are essential for the assembly of an exopolysaccharides-rich matrix and the development of cospecies biofilms. The synergism between *C*. *albicans* and *S*. *mutans* can enhance the virulence of mixed biofilms formed on tooth surfaces and contribute for the severity of early childhood caries and other polymicrobial biofilm infections [23,25].

On the other hand, in the scanning electron microscopy analyses, we noticed that the presence of *S*. *mutans* cells caused a reduction in the hyphae formation in the mixed biofilms composed by *C*. *albicans* and *S*. *mutans*. Similar results have been reported in the study of Pereira-Cenci et al. [5], in which the presence of *S*. *mutans* cells in the biofilm increased *C*. *albicans* growth. However, using confocal laser scanning microscopy, these authors observed that *S*. *mutans* suppressed hyphae formation by *C*. *albicans* and emphasized that the effects of *S*. *mutans* on *C*. *albicans* filamentation should be considered in studies on the prevention of oral candidiasis.

In order to investigate how *S*. *mutans* affects *C*. *albicans*, we also tested the effects of *S*. *mutans* culture filtrate (without *S*. *mutans* cells) on *C*. *albicans* biofilms. *S*. *mutans* culture filtrate was able to inhibit the CFU/mL number of *C*. *albicans* and block the hyphae formation, with total absence of filamentation in the biofilms analyzed by SEM. After that, we performed another experiment focused in the *C*. *albicans* filamentation. The hyphae formation was induced *in vitro* by incubation of *C*. *albicans* with fetal bovine serum. The presence of *S*. *mutans* cells was not capable to inhibit hyphae formation by *C*. *albicans*. Possibly, *S*. *mutans* cells did not affect *C*. *albicans* morphogenesis because *S*. *mutans* was placed in the plates with only bovine serum (without culture medium), in which these microorganisms were unable to grow and produce acids or bacterial secreted molecules that could inhibit *C*. *albicans*. On the other hand, *S*. *mutans* culture filtrate, that probably contained acids or other metabolites, was capable to inhibit *C*. *albicans* filamentation.

Then, the presence of acids in the *S*. *mutans* supernatant was evaluated by pH measurement. It has been reported that a neutral to alkaline pH favors the growth of hyphae, while an acidic pH has a inhibitory effect on filamentation [26]. The *S*. *mutans* supernatant and the BHI broth (Control group) showed similar pH values, represented respectively by 7.0 and 7.3. These findings indicate that *C*. *albicans* filamentation was not affected by pH in the culture medium. Jarosz et al. [1] investigated the interaction between *C*. *albicans* and *S*. *mutans* based on production of secreted molecules. They tested the effect of spent medium of *S*. *mutans* on *C*. *albicans* germ-tube formation at different phases of *S*. *mutans* growth (4, 6, 8 and 24 h cultures). Only *S*. *mutans* spent medium of 4 h culture was capable to inhibit germ-tube formation, indicating that *S*. *mutans* secretes quorum sensing molecule during the early stages of growth that inhibits the *C*. *albicans* morphological transition.

These results suggest that, when cultured *in vitro*, *S*. *mutans* can produce signaling molecules with antifungal activity that inhibit the growth of *C*. *albicans* cells and mainly block its filamentation capacity. Then, the *in vitro* results were investigated in more detail through *in vivo* studies, using *G*. *mellonella* as a model host of *Candida* infection. This is the first study that attempts to investigate the effects of *S*. *mutans* on the pathogenicity of *C*. *albicans* and development of experimental candidiasis in animal models. Initially, we performed an experiment to elucidate the host response of *G*. *mellonella* to mono-infection by *S*. *mutans*. We found that *S*. *mutans* cells were capable of infecting and killing *G*. *mellonella* larvae. The increasing concentrations of the *S*. *mutans* cell number resulted in gradually decreasing survival of the infected larvae and 10^5^ cells/larva was defined as a sublethal concentration. Abranches et al. [27] also injected *S*. *mutans* in *G*. *mellonella* larvae and demonstrated that *G*. *mellonella* can be a suitable model to study the virulence potential of *S*. *mutans* strains.

Once the sublethal concentration of *S*. *mutans* was defined, the microbial interaction between *C*. *albicans* and *S*. *mutans* was investigated in the *G*. *mellonella* model. The results from survival analysis showed that the injection with *S*. *mutans* cells or *S*. *mutans* filtrate culture prolonged the survival rate of the larvae infected by *C*. *albicans* when compared to the control groups, suggesting that *S*. *mutans* reduced the pathogenicity of *C*. *albicans* in these animals.

Since in this study, *G*. *mellonella* larvae were inoculated with a known quantity of *C*. *albicans* by injecting the fungal cells directly into the hemocoel, we also evaluated the effects of *S*. *mutans* on *C*. *albicans* cells present in the hemolymph of *G*. *mellonella* at different times of infection. The results showed that *S*. *mutans* cells and *S*. *mutans* culture filtrate did not affect the number of *C*. *albicans* in the hemolymph. The changes in the *C*. *albicans* CFU/mL occurred only by the action of immune system. The number of *C*. *albicans* cells recovered immediately after inoculation (0 h) did not differ from the fungal load inoculated; however, a reduction in the number of recovered cells was observed at the subsequent time after inoculation (8 h), suggesting that the larval immune system was able to combat infection with *C*. *albicans*, reducing the number of *Candida* CFU/mL at these times of infection. After this period, *C*. *albicans* was able to overcome the immune system of *G*. *mellonella*, reaching its peak proliferation (12 h) and a fungal load similar to that inoculated at the beginning of the test.

In addition to CFU/mL count of *C*. *albicans* in the hemolymph of *G*. *mellonella*, the experimental candidiasis can be evaluated by histological analysis. It has been described that *C*. *albicans* is capable to form hyphae in the *G*. *mellonella* tissues during the infection process. Therefore, analysis of internal structures can be used to observe the fungal state and identify forms of yeast and hyphae [7]. In this study, histological analysis of the fat body and other internal structures of *G*. *mellonella* infected with *C*. *albicans* showed a significant reduction in the presence of hyphae when *C*. *albicans* was associated with the *S*. *mutans* supernatant, confirming the results of the *in vitro* tests. These results suggest that *S*. *mutans* may secrete subproducts into the culture medium *in vitro* that are able to inhibit the morphological transition of *C*. *albicans in vivo*.

Although there is an abundance of published reports illustrating the extent to which bacteria and fungi are capable of interacting with other microorganisms within their vicinity, the majority of biomolecules responsible for influencing these biological processes remain unknown [28]. Recently, some studies were developed to characterize biomolecules excreted by *S*. *mutans* into the extracellular environment. Joyner et al. [6] described the structural features of the major putative hybrid polyketide synthase-nonribosomal peptide synthetase (PKS-NRPS) derived metabolite from *S*. *mutans* UA159. This biomolecule, which was dubbed the mutanobactin, was capable to suppress the morphological transition of *C*. *albicans* from yeast to hyphae. Vílchez et al. [29] identified another compound secreted by *S*. *mutans*, trans-2-decenoic acid, which suppressed morphogenesis at concentrations that do not affect fungal growth. Zvanych et al. [30] tested whether mutanobactins display any immunomodulatory activity using *in vitro* models of macrophage cell line. Interestingly, mutanobactin B caused a significant increase in pro-inflammatory cytokines, such as IL-6 and Il-12, suggesting that mutanobactins may play an important role in modulating immune response.

In summary, the results obtained showed that the *S*. *mutans* culture filtrate exerted inhibitory effects on the biofilm formation, morphogenesis and pathogenicity of *C*. *albicans*, attenuating the experimental candidiasis in animal models. These results will certainly contribute to the development of new therapeutic strategies for human candidiasis. However, the exact mechanism whereby *S*. *mutans* interferes with morphogenesis and pathogenicity of *C*. *albicans* still needs to be elucidated. Therefore, comprehensive studies involving molecular and immunological assays are needed to better understand how oral bacteria *S*. *mutans* can modulate the pathogenicity of *C*. *albicans*.

## Supporting Information

S1 Fig Susceptibility of *G*. *mellonella* to infection with *S*. *mutans*. Survival curves of *G*. *mellonella* larvae inoculated with different concentrations of *S*. *mutans* (10^4^ to 10^7^ cells/larva) to determine the sublethal concentration of this microorganism.
(TIFF)

S2 Fig Number of fungal cells in *G*. *mellonella* hemolymph after infection with *C*. *albicans*. The number of cells was quantified in hemolymph pools of three larvae per time point after infection. Tukey test, *P* ≤ 0.05.
(TIF)
