# Supplementary figures and images for: Streptococcus mutans Can Modulate Biofilm Formation and Attenuate the Virulence of Candida albicans
Source: PLoS One. 2016 Mar 2;11(3):e0150457. doi: 10.1371/journal.pone.0150457 (PMC4774980; doi:10.1371/journal.pone.0150457)

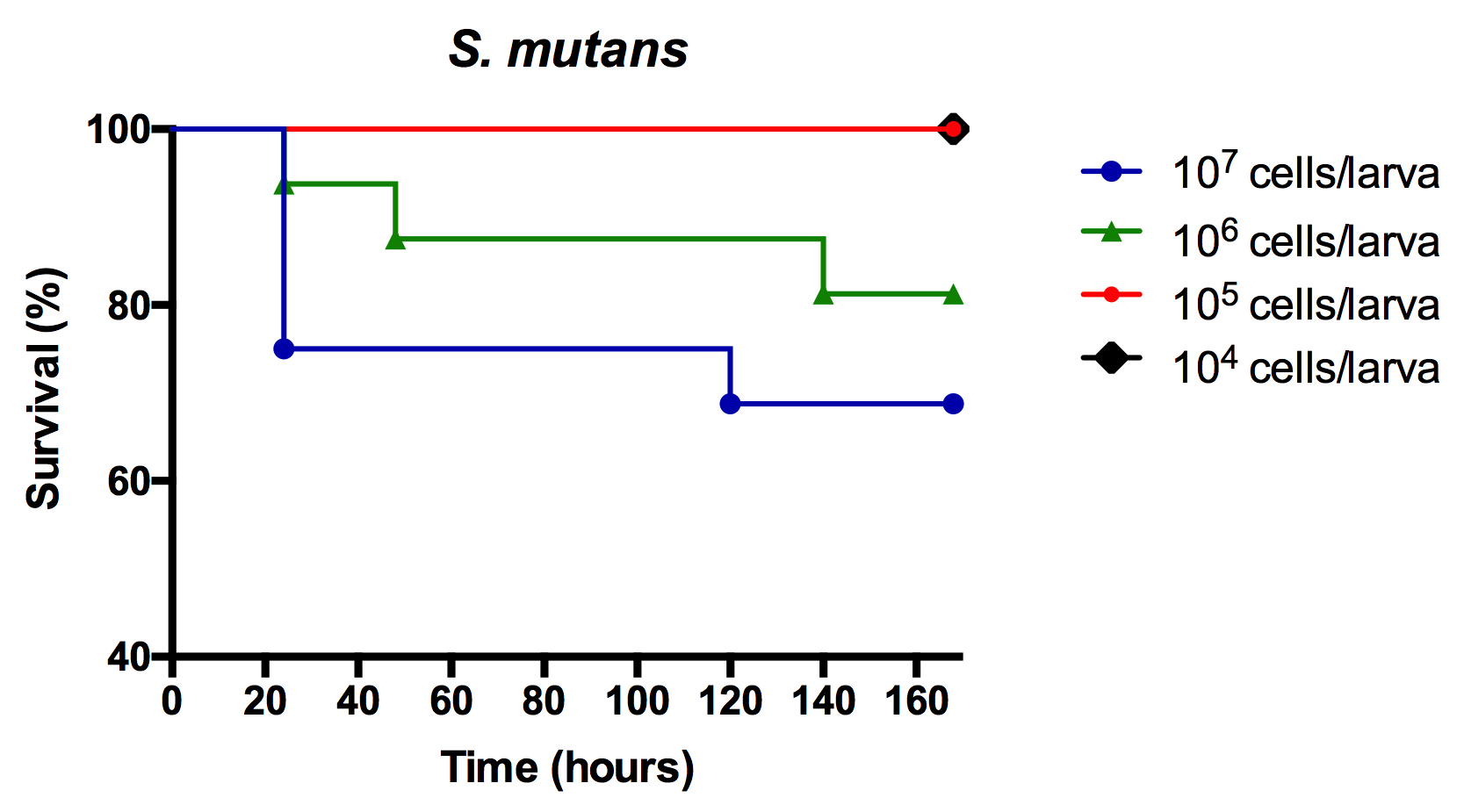

Supplement: S1 Fig — Survival curves of G. mellonella larvae inoculated with different concentrations of S. mutans (104 to 107 cells/larva) to determine the sublethal concentration of this microorganism. (TIFF) [file pone.0150457.s001.tiff]

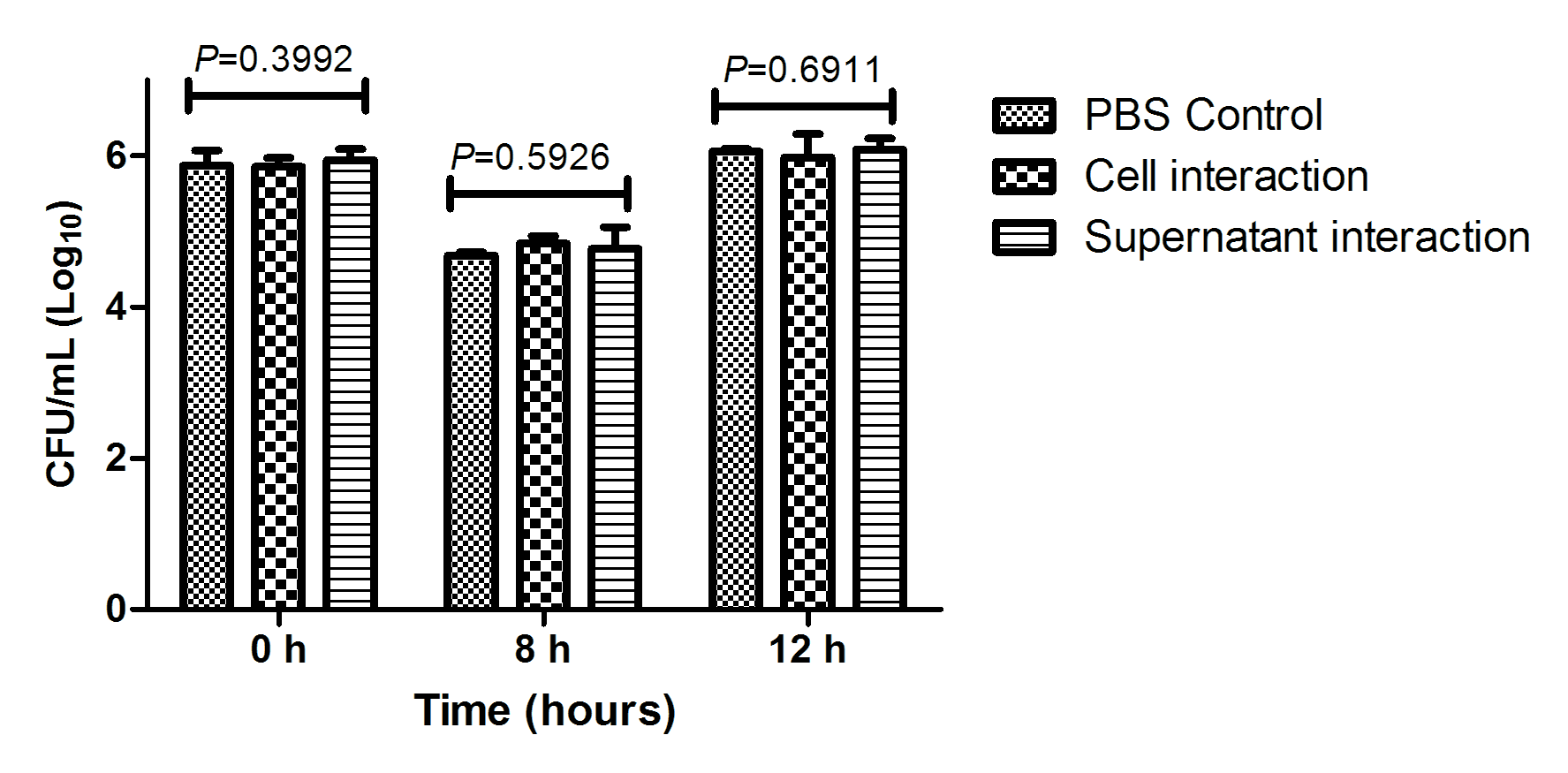

Supplement: S2 Fig — The number of cells was quantified in hemolymph pools of three larvae per time point after infection. Tukey test, P ≤ 0.05. (TIF) [file pone.0150457.s002.tif]
